# Use of Lipid Extract of Oat Flour as a Peroxygenase-Containing Biocatalyst Active in Organic Solvents

**DOI:** 10.3390/ijms26199431

**Published:** 2025-09-26

**Authors:** Claudia Sanfilippo, Angela Patti

**Affiliations:** Institute of Biomolecular Chemistry, National Research Council of Italy, Via Paolo Gaifami 18, 95126 Catania, Italy; claudia.sanfilippo@cnr.it

**Keywords:** peroxygenase, oat flour, lipid fraction, epoxidation, thioanisole oxidation, organic solvent

## Abstract

Oat seeds contain peroxygenase, a heme enzyme localized in both the microsomal and the lipid droplet fractions, which are usually separated by multiple ultracentrifugation steps. In this work, it was shown that peroxygenase activity is retained in the lipid fraction (LF) of oat seeds, which is obtained by simple extraction of flour with organic solvents. The enzymatic activity of this crude preparation was tested in the oxidation of thioanisole in comparison with a peroxygenase-containing microsome preparation (MP) from the same plant source in both aqueous medium and pure organic solvents. In most cases, higher activity was observed in the LF preparation, which proved to be stable in organic solvents for at least 24 h, thus offering a good option for the oxidation of highly water-insoluble substrates. LF-peroxygenase maintained the same stereoselectivity features observed with MP, as also demonstrated in the epoxidation reaction of limonene. A protocol for the oxidation of thioanisole in CH_3_CN was set up on a preparative scale, and the corresponding sulfoxide was obtained at concentration of 1.7 M and with 84% *ee*.

## 1. Introduction

Biocatalyzed oxidations have attracted growing interest as a sustainable alternative to chemical processes, which often involve toxic metal catalysts and harsh conditions. This approach is facilitated by the use of benign oxidants such as O_2_ or H_2_O_2_ and the intrinsic ability of enzymes to provide chemo- and stereoselectivity [1,2]. Since the cofactor (NADH or NADPH) dependence for the activity of most oxidases may negatively impact the economics of the process, great efforts are currently focused on the optimization of cofactor regeneration methods [3]. A promising and alternative approach relies on the use of H_2_O_2_-dependent enzymes [4], which have shown great potential in organic synthesis, owing to the large portfolio of catalyzed reactions, as well as in bioremediation processes [5].

Peroxygenases (EC 1.11.2) are heme-containing enzymes able to catalyze the oxyfunctionalization of different substrates by using peroxides (H_2_O_2_ or R-OOH) as a source of oxygen, without the need for auxiliary flavoproteins or nicotinamide cofactors. Among these enzymes, which are widely distributed in living organisms, unspecific peroxygenases (UPOs, EC 1.11.2.1) from fungi have been the most investigated for their potential in biocatalyzed organic synthesis and the possibility to improve their activity and robustness by direct evolution techniques [6,7,8]. Furthermore, peroxygenases are able to stereoselectively insert an oxygen atom onto C-H carbon atoms, ethylenic systems or heteroatoms, and are therefore of interest in the synthesis of chiral compounds [9].

Plant peroxygenases (EC 1.11.2.3), ubiquitously present in land plants, play an important physiological role in the oxidative metabolism of fatty acids and oxylipins [10] and are also involved in the plants’ response to abiotic stresses [11] and pathogens [12]. Most interest has been focused on oat seed peroxygenase, which has been structurally characterized as a caleosin due to the presence of a calcium-binding EF-hand motif, and it is localized in both microsomal and lipid droplet fractions of the seeds [13]. Although oat seed peroxygenase has been obtained in purified form, it is rather unstable, and the catalytic activity of the enzyme in the epoxidation of polyunsaturated fatty acids has been evaluated using the microsome fraction as a whole, after separating it from the other intracellular components by ultracentrifugation [14,15].

More recently, we developed an easy protocol to obtain a crude and stable preparation of microsomal oat seed peroxygenase [16] and tested it in the regioselective epoxidation of ethanolamides of eicosapentaenoic (EPA) and docosaesanoic (DHA) acids [17]. The same enzymatic preparation was then shown to be an active catalyst in the stereospecific epoxidation of limonene [18], in the asymmetric oxidation of aromatic sulfides [19] and in the stereoselective oxidation of symmetric 1,3-dithianes and *bis*-(phenylthio)alkanes [20].

Using plant whole tissues or plant extracts as biocatalysts is considered a promising approach in the development of green organic synthesis, for the intrinsic advantages of wide availability of the starting material at low cost, easy disposal after use and biodegradability. Different vegetables have been tested for their lipase activity in the stereoselective hydrolysis of labile organic compounds [21], while *Daucus carrota* roots have been shown to be active in the reduction of different classes of ketones to produce chiral alcohols with excellent enantioselectivity [22]. Peroxidase activity in onion solid waste and horseradish roots has been exploited for the synthesis of fused heterocycles [23,24], and bioremediation applications have also been demonstrated for different types of peroxidase-containing biowaste [25,26]. In this context, oat represents the most investigated source of peroxygenase, and the study of the enzyme activity has been, up to now, focused on the microsomal fraction of the seeds.

It is known that oat seed peroxygenase is also contained in the lipid droplet fraction of the seeds [13], but this sub-cellular fraction has not been considered a potential source of the enzyme. Lipid droplets are organelles present in all eukaryotic cells, composed by a core of triglycerides surrounded by a phospholipid layer in which a variety of proteins can be tightly embedded. They have long been considered a mere reservoir of lipids, but it is now accepted that, thanks to the proteins associated with them, they play important roles in lipid regulation and metabolism [27,28,29]. Lipid droplets are usually collected as a floating layer after ultracentrifugation of aqueous solutions of lysed cells [30], while methods based on extraction with organic solvents have been less frequently reported [31].

In our protocol for crude microsomal peroxygenase, oat flour was preliminarily defatted by diethyl ether extraction, and the organic phase discarded, although it may be worth examining this phase for its residual enzymatic activity. As part of our study on the development of crude enzymatic preparations from plants [16,17,18,19,20,32] and their application in organic synthesis, we planned to study the catalytic activity of the lipid fraction (LF) of oat seeds as an economical and easily accessible alternative to the microsomal enzymatic preparation (MP) from the same plant source.

## 2. Results

### 2.1. Composition of Lipid Fraction (LF) of Oat Seed

Oat oil has a unique composition among cereals due to its significant content (about 30%) of phospholipids and glycolipids in addition to the typical triglycerides [33]. Several protocols have been reported for the extraction of oat oil from seeds, and in some cases, a fractionation between triglycerides and polar lipids can be obtained. Common procedures require solvent extraction for a long time under heating [34], but efficient methods based on supercritical carbon dioxide have been recently developed [35].

At the onset of our work, we tested three different solvents for the extraction of LF from ground seeds, avoiding mixtures with alcohols, which are known to deactivate enzymes. In parallel analyses, we treated oat seeds with *n*-hexane, diethyl ether or acetone, then the organic phase was separated by centrifugation and taken to dryness. In all cases, LF was obtained in the range 3.5–4% (*w*/*w*) as a yellow-brown oil (Figure S1) and no significant changes were observed in its fatty acid composition, which was determined by ^1^H-NMR spectroscopy applying a reported methodology [36,37].

The LF was mainly composed by fatty acid triglycerides, along with some fatty acids in free form, while partially esterified glycerol derivatives (i.e., mono-acyl and di-acyl glycerols) were below the detection limit of the ^1^H-NMR technique. Free fatty acids may be formed by the action of lipase on triglycerides once the seeds are broken, as their content was found to increase in the extraction of not freshly ground seeds.

The analysis of ^1^H-NMR spectra (Figure S2) revealed an approximate 1:1 ratio (36 ± 2%) between mono-unsaturated (oleic) and di-unsaturated (linoleic) acyl chains, with the remaining part consisting of saturated (mainly palmitic) and <4% of tri-unsaturated (linolenic) acyl chains. Data in the literature show some variability in the results [34,38], depending on the considered oat specie, the analysis method and the extraction procedure, but all findings highlight evidence of the high content of unsaturated fatty acids, which makes oat oil very attractive in cosmetics and food preparation [33].

The peroxygenase activity of LF, assessed by ABTS assay and confirmed by oxidation of thioanisole, showed that extraction of oat seeds with acetone resulted in a loss of enzymatic activity. In contrast, comparable levels of activity were detected in the *n*-hexane and diethyl ether extracts (Figure S3).

We therefore chose to use diethyl ether as extraction solvent for further studies on LF from oat seeds. Light microscopy images of LF suspended in buffer showed the presence of structured oil bodies, which tend to aggregate below pH 7.5 (Figure S4) with a behavior consistent with data in the literature [39].

### 2.2. Peroxygenase Activity of Oat LF: Sulfoxidation Reaction

The enzymatic activity of LF-peroxygenase was preliminarily assessed by ABTS assay and monitored over time. The results showed that the enzyme remained stable for at least up to 4 weeks in phosphate buffer solution and up to 6 months when stored as an oil at 4 °C (Figure S5). By the same assay, no significant variations were detected in the enzymatic activity of LF preparations derived from different commercial brands of oat seeds or from different extraction batches of the same plant source.

**Scheme 1:**
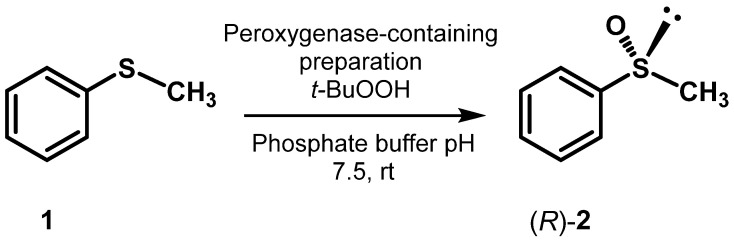
Oxidation of thioanisole catalyzed by peroxygenase-containing preparations from oat flour.

While the ABTS assay was used for relative measurements, the activity of enzymatic preparations was determined from reaction rates in the oxidation of thioanisole, **1** (Scheme 1). Under standard conditions (phosphate buffer pH 7.5, *tert*-butyl hydroperoxide, TBHP, as oxidant, 30 °C), LF exhibited higher enzyme activity/mg preparation compared to the MP from the same flour, but in both cases, sulfoxide product **2** was obtained with (*R*)-absolute configuration and the same enantiomeric excess (79 ± 1% *ee*).

Hanano et al. observed higher enzymatic specific activity for peroxygenase purified from lipid droplets compared to that isolated from oat microsomes [13], but we have no evidence to support this claim, and the higher peroxygenase activity/mg sample may simply be due to the higher concentration of the enzyme in this crude LF preparation compared to MP. Furthermore, in the same study, the same structure was demonstrated for the enzyme present in the two cellular compartments, as could also be deduced from the same stereopreference and levels of stereoselectivity observed for both the LF and MP preparations.

**Figure 1:**
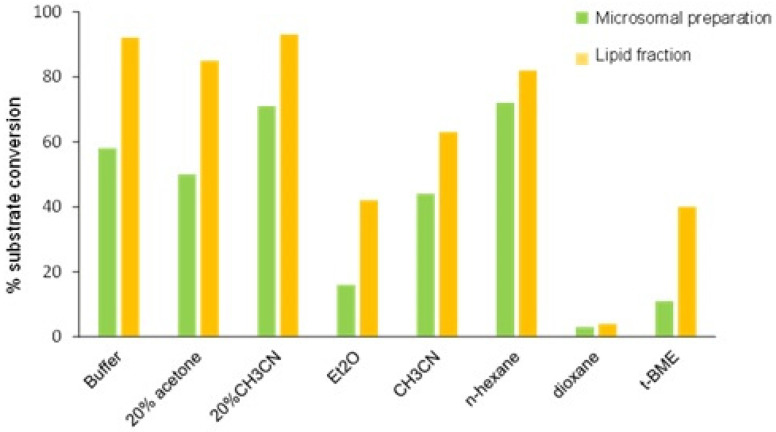
Oxidation of thioanisole catalyzed by peroxygenase-containing preparations from oat flour in different solvents. Conditions: enzymatic preparation (10 mg), thioanisole (21 mg, 0.17 mmol), TBHP (0.20 mmol), solvent (2.5 mL), 30 °C, 20 min.

The catalytic activity of LF-peroxygenase was not significantly affected by the presence of cosolvent (20% acetone or CH_3_CN) in the reaction medium, unlike what was observed for MP, which increased its activity in the buffer/CH_3_CN system (Figure 1).

Considering that the LF was separated by treatment with diethyl ether, one might expect that the catalytic activity would be retained and displayed in pure organic solvents. Some organic solvents with different polarity were then considered as reaction medium, and in all the cases, higher conversion of **1** was detected in reactions catalyzed by LF compared to MP. This result seems to suggest a role of the lipid matrix in enhancing the solubilization of the enzyme in the reaction medium and/or protecting it from potential denaturation induced by the organic solvent.

Coating enzymes with lipids, indeed, has been shown to be a valuable approach for increasing the solubility of different enzymes (lipases, glycosidases, horseradish peroxidase) in organic solvents, thereby facilitating their use in non-aqueous medium or in two-phase systems [40], and positive effects on enantioselectivity and activity also have been reported [41,42,43]. Given that the formation of lipid-coated enzymes is driven by spontaneous non-covalent interactions, usually achieved by simply “mixing and stirring” the components, the peroxygenase contained in the lipid fraction could be viewed as a “naturally coated” peroxygenase by means of the lipids present in the oat seeds.

Data in Figure 1 show that for both LF and MP, *n*-hexane provided the best alternative to an aqueous medium, while lower enzymatic activity was observed in more polar solvents. These results, consistent with those obtained in the epoxidation of linoleic acid with peroxygenase from oat microsomes [13], confirm the broad solvent tolerance of the enzyme and offer a strategy for increasing productivity in the biotransformation of highly water-insoluble substrates. Moving from buffer to aqueous solvents, the stereoselectivity in the oxidation of the sulfur atom remained unchanged, while a slightly increased enantiomeric excess of (*R*)-**2** (80–84% *ee*) was measured in reactions performed in pure organic solvents.

Organic solvents in which LF-peroxygenase showed the best reactivity (Et_2_O, CH_3_CN and *n*-hexane) were chosen for further stability studies. The stability of the enzyme was assessed by pre-incubation experiments in which the enzymatic preparation was allowed to stand in the solvent of choice for 20 h and then used as biocatalyst in the oxidation of **1** by adding the reagents. In the first series of experiments, the solvent was removed after incubation, and the enzyme was reacted in phosphate buffer in parallel with a freshly prepared reaction mixture. Noteworthily, the enzymatic activity was completely retained (Figure S6, red box), suggesting that treatment with organic solvents does not cause any irreversible deactivation and that any possible protein unfolding could be considered reversible, with a restoration of the active conformation of the enzyme in phosphate buffer.

In another set of experiments, the LF-peroxygenase was left to react in the pre-incubation solvent, and in this case, a slight decrease (12–15%) in activity was observed compared to the reference reaction with fresh enzymatic preparation in the same solvent (Figure S6). These results suggest that the reduction in enzyme activity observed in pure organic solvents compared to aqueous buffer is not due to enzyme deactivation, but it rather stems from the effect of the solvent on the active conformation of the enzyme and/or on the ability of reagents to approach the active site of the enzyme.

The significance of these results becomes apparent when compared with those of unspecific fungal peroxygenases, which are mostly used in aqueous solvents and require immobilization to retain their activity in neat organic solvents [44,45]. In a stability study of *r*AaeUPO from *Agrocybe aegerita* [46], the residual activity of the enzyme pre-incubated in buffer containing 50% or 80% CH_3_CN decreased to 50% and 1%, respectively, after just 1 h of exposure to the organic solvent mixtures, while it did not change significantly in the absence of CH_3_CN over 150 h. For 50% CH_3_CN/buffer solvent, the authors attributed the observed decrease in activity to a partial precipitation of the enzyme in the incubation medium, but in any case, no activity was detected in pure CH_3_CN.

### 2.3. Peroxygenase Activity of Oat LF: Epoxidation Reaction

Epoxidation of unsaturated fatty acids, which is the physiological reaction catalyzed by oat seed peroxygenase, has been reported for the enzyme localized in microsomes, and in our previous work, we have shown that efficient catalysis also can be achieved using a crude MP of the enzyme [16,17,18]. The same reaction with a peroxygenase from lipid droplets has been reported only with the purified enzyme, and the alternative use of a crude lipid fraction might be hampered by the presence of triglycerides in the matrix, which themselves can undergo epoxidation and compete with the substrate. While competitive epoxidation of triglycerides does not pose issues in the sulfoxidation reaction, which occurs at a much higher rate [13], it must be taken into account when dealing with substrates containing C-C double bonds.

Preliminary experiments indicated that treatment of LF with TBHP resulted in the epoxidation of about 40% of the fatty matrix in 4 h in aqueous buffer or CH_3_CN (Figure S7), while the same reaction in *n*-hexane or diethyl ether occurred at an approximately three-fold lower rate. Epoxidation of LF was monitored by the decrease in the signal for ethylene protons in the NMR spectrum (Figure S8) and the appearance of new resonances for the epoxide moiety. It was found that epoxidation is favored by the slow addition of TBHP, as might be expected from the known deactivation of the enzyme by oxidants.

**Scheme 2:**
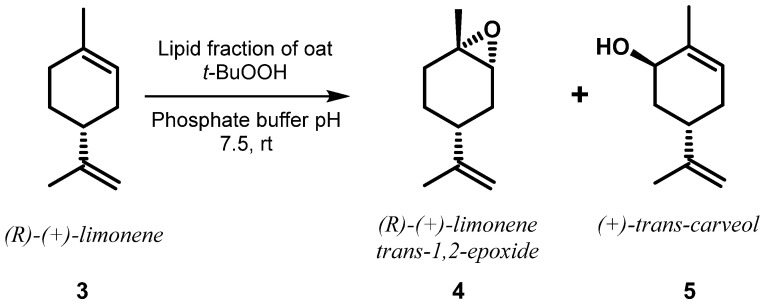
Oxidation of limonene catalyzed by peroxygenase-containing LF of oat flour.

The LF-catalyzed epoxidation of limonene **3** was tested in different solvents using an excess of TBHP to compensate for the oxidant consumption due to the occurrence of a parallel epoxidation of the fatty matrix. In phosphate buffer or CH_3_CN, effective substrate conversion (83% and 51%, respectively) was observed in 2 h, while the same reaction in Et_2_O or *n*-hexane proceeded at a significantly lower reaction rate (6% and <5%, respectively). In phosphate buffer, prolonging the reaction time to 4 h resulted in full conversion of **3**, accompanied by some decrease (−24%) in the amount of double bonds present in the triglyceride matrix. The oxidation proceeded with excellent diastereoselectivity, giving the *cis*-1,2-epoxide **4** as main product (*dr* 99:1) together with a low amount (5%) of carveole **5** as single diastereomer (Scheme 2).

### 2.4. Increase in Productivity in LF-Catalyzed Oxidation of Thioanisole

The use of aqueous solvents in peroxygenase-catalyzed reactions ensures high efficiency but, on the other hand, could represent a limit for productivity, due to the low solubility of most of the organic substrates in the reaction medium. Although some reactions have been optimized in two-phase systems, a strategy applied to date only to liquid substrates that constitute one of the two phases, peroxygenase catalysis in non-aqueous media has been little investigated.

Recently, Zhang and coworkers developed a solvent-free procedure for oxidation of **1** using an immobilized fungal peroxygenase, but in the case of solid sulfides, they were forced to use a cosolvent and concluded that, at the current stage, a solvent-free reaction exhibiting incomplete conversion is less advantageous as compared to one using a cosolvent but enabling full conversion [45].

Given the reactivity of LF in organic medium, we tried to improve productivity in the oxidation of **1** by suitable adjustment of the substrate:enzyme ratio and addition rate of TBHP. Under standard conditions, sulfide **1** at 70 mM concentration was completely oxidized within 1 h in both CH_3_CN or *n*-hexane, and we used these solvents in the scale-up of the process.

**Figure 2:**
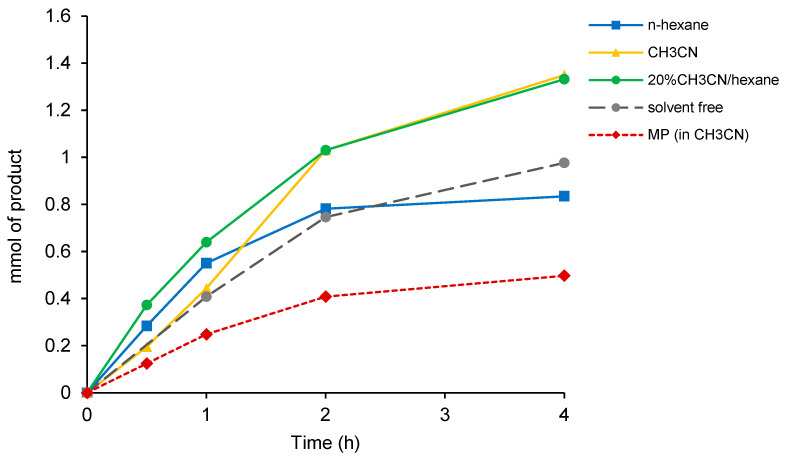
Preparative reactions of oxidation of thioanisole in different conditions. Conditions: enzymatic preparation (40 mg), thioanisole (220 mg, 1.77 mmol), TBHP (300 μL, 1.2 eqv., 125 μL/h), solvent (2.2 mL), 30 °C.

To a solution of **1** at initial 0.7 M concentration, the oxidant was added at 125 μL/h (1.2 eqv.), and the substrate conversion was monitored by HPLC. Parallel reactions were carried out in *n*-hexane, CH_3_CN and in *n*-hexane:CH_3_CN 80:20 mixture. In the latter two solvents, about 75% substrate conversion was reached after full addition of oxidant (2.5 h) and slightly increased after prolonging the reaction time up to 24 h, while a lower reaction rate was observed in *n*-hexane. Decreasing the addition rate of TBHP to 80 μL/h resulted in unchanged reaction rate profiles, and in all the cases, sulfoxide (*R*)-**2** was formed as an almost exclusive product (<5% of sulfone was detected in the reaction mixture) with good enantiomeric purity (85% *ee*) (Figure 2).

When **1** was used as both substrate and reaction medium, the biocatalyzed oxidation proceeded with the same initial reaction rate as in *n*-hexane, but the optical purity of the corresponding sulfoxide dropped to 74% *ee*. It should be noted that, as the reaction time increases, the reaction medium becomes mainly composed of water, along with unreacted thioanisole and *tert*-BuOH, resulting from the reduction of oxidant.

For the sake of comparison, a parallel reaction in CH_3_CN was carried out using MP as biocatalyst, and a sensibly reduced reaction rate, coupled to incomplete substrate conversion even after a long time, was observed. This finding seems to indicate that peroxygenase in microsomes is more susceptible to peroxide-induced deactivation compared to the same enzyme entrapped in lipid matrix.

The reaction was also set up in continuous mode by simultaneously adding two solutions of **1** and TBHP (1:1.2 molar concentration ratio) to a solution of LF-peroxygenase in CH_3_CN. Under these conditions the substrate conversion remained constant at 93% for up to 4 h, then decreased in the following hours, suggesting that the unconsumed oxidant gradually erodes the enzyme activity. At an addition rate of 427 μmol/h of **1**, substrate conversion reached 75% over 8 h, and sulfoxide **2** was obtained in 70% isolated yield (2.4 mmol, 1.7 M final concentration) and 84% *ee*, without any interference from the lipid matrix during chromatographic purification.

## 3. Materials and Methods

### 3.1. General

Thioanisole, limonene, **3**, and 2,2′-azino-bis(3-ethylbenzothiazoline-6-sulfonic acid) (ABTS) were purchased from Aldrich (Merck LifeScience, Milan, Italy) and used as received. Aqueous *tert*-butyl hydroperoxide (TBHP, 70 wt% in H_2_O, 7.3 M) was obtained from Santa Cruz Biotechnology, Dallas, TX, USA. Potassium phosphate buffers (50 mM) were prepared from K_2_HPO_4_, adjusting pH to 7.5 or 5.5 with H_3_PO_4_.

Commercial seeds of air-dried oat (*Avena sativa*) from organic crops were finely ground by a POWTEQ (Fulltech Instruments, Roma, Italy) Micro Ball Mill GT300 using 50 mL steel grinding jars and spheres (Ø 30 mm) by means horizontal oscillations at high frequency. The microsomal preparation (MP) from oat seeds was obtained as previously reported [19] and stored as lyophilized powder at −18 °C.

Suspensions of LF in phosphate buffer (pH 7.5 or 6.5) were examined with a phase-contrast inverted AXIOVERT 100 microscope (Zeiss, Jena, Germany), capturing the image with an Axiovision imaging system.

TLC analyses were performed on aluminum plates coated with silica gel and fluorescent indicator F254, revealing the compounds by UV. Column chromatography was performed on Silica gel 60 (Merck, 40–63 μm) using the specified eluents. ^1^H-NMR and ^13^C-NMR spectra were recorded with a Bruker Avance^TM^ 400 (Bruker Italia, Milano, Italy) spectrometer at 400.13 and 100.62 MHz, respectively.

HPLC analyses were carried out on a Dionex (ThermoFisher Scientific, Milan, Italy) instrument equipped with an Ultimate 3000 high-pressure binary pump, an ASI-100 autosampler, a TCC-100 thermostated column compartment and a UVD-100 multiple wavelength detector set at 220 nm. Chromeleon software (version 6.7) was used for instrument control, data acquisition and data handling. Chiral HPLC was performed on a Phenomenex (Phenomenex, Inc., Bologna, Italy) Lux 5 μm Cellulose-1 (250 × 4.6 mm) column eluting with *n*-hexane/EtOH 90:10 at a flow rate of 0.5 mL/min.

GC analyses were performed on a fast GC Shimadzu 17-A instrument (Shimadzu Italia srl, Milan, Italy), equipped with a flame ionization detector and a Supelco (Merck LifeScience, Milan, Italy) SPB-5 capillary column (15 m × 0.1 mm ID × 0.1 μm film thickness). For the analyses, the following parameters were set: N_2_ flow rate 0.9 mL/min, injector temperature 250 °C, detector temperature 280 °C. The oven temperature was held at 60 °C for 1 min, then raised to 280 °C at 10 °C/min.

UV measurements were carried out on a UV–vis Agilent 8453 diode spectrophotometer (Agilent Technologies Italia, Milan, Italy).

### 3.2. Extraction of Lipid Fraction (LF) from Oat Seed

In a typical procedure, oat seeds (84 g) were ground with a vibro-mill, applying 2 cycles of 1 min each at a speed of 1500 rpm. To the obtained flour, Et_2_O (or other tested solvent, 150 mL) was added, and the suspension stirred for 5 min at room temperature, then centrifuged at 4000 rpm (2930× *g*) for 20 min. The supernatant was separated, and the procedure repeated two more times on the residue, suspended in fresh organic solvent (2 × 80 mL). Pooled supernatant fractions were taken to dryness under vacuum at 30 °C to give the lipid fraction (LF) as a yellow-brown oil (3.5–4.0% *w*/*w* yield), while the pellets were used to obtain microsomal preparation (MP). The lipid mixture composition was determined by ^1^H-NMR spectrum in CDCl_3_ (Figure S2), while morphology was visualized by light microscopy (Figure S4). The LF fraction was stored in the refrigerator at 4–8 °C and was shown to be stable for up to 6 months.

### 3.3. Microsomal Preparation (MP) from Defatted Oat Flour

The pellet obtained in the above-described defatting of oat flour was dried overnight at room temperature to give a powder (80 g) that was suspended in distilled water (80 mL) and left to stir for 5 min at room temperature. The slurry was centrifuged at 4000 rpm (2930× *g*) for 7 min, and the supernatant was separated. The pellet was washed with water, and the suspension centrifuged, repeating the procedure two times. The pooled supernatant fractions were freeze-dried to give 5.5 g (6.9% *w*/*w* yield) of a light white powder (microsome preparation, MP).

### 3.4. ABTS Assay

A suspension of enzymatic preparation in phosphate buffer pH 7.5 (100 μL, 5 mg/mL) was diluted with 2.5 mL of phosphate buffer at pH 5.5 in a UV cuvette (1 × 1 cm) and left to stir at 30 °C for 10 min. A solution of ABTS in phosphate buffer pH 5.5 (200 μL, 16.4 mg/mL, 0.0064 mmol) was added, and the reaction started by addition of 73 mM TBHP (200 μL, 0.0146 mmol). The reaction progress was monitored by measuring the increase in absorbance at 418 (ε 36 mM^−1^ cm^−1^) for 10 min at room temperature.

### 3.5. Determination of Enzymatic Activity

LF (2.5 mg) or MP (5.4 mg) was added to phosphate buffer at pH 7.5 (2.5 mL) and stirred for 10 min at 30 °C. To the suspension, thioanisole **1** (30 μL, 0.25 mmol) and TBHP (26 μL, 0.19 mmol) were added, and the reaction maintained at 30 °C under vigorous magnetic stirring. For monitoring substrate conversion, aliquots (0.2 mL) of the reaction mixture were extracted with Et_2_O/MeOH 9:1 *v*/*v* (0.5 mL), and the organic phase dried on Na_2_SO_4_ and analyzed by HPLC. Enzyme activities of 1.97 and 0.86 μmol/mg/min were determined for LF and MP, respectively.

### 3.6. General Procedure for Oxidation of ***1***

To a suspension of enzymatic preparation, LF or MP (10 mg), in 50 mM phosphate buffer at pH 7.5 or in the solvent of choice (2.5 mL) compound **1** (21 mg, 0.17 mmol), was added, and the mixture vigorously stirred at 30 °C for 10 min. The reaction was started by adding TBHP (28 μL, 0.2 mmol) within 10 min. At a suitable time, aliquots (0.2 mL) of the reaction mixtures were withdrawn and extracted with Et_2_O/MeOH 9:1 *v*/*v* (0.5 mL) as above to be analyzed by HPLC.

### 3.7. Evaluation of the Stability of LF-Peroxygenase in Organic Solvents by Pre-Incubation Experiments

Two series of experiments were carried out according to the procedures described below.

Series A: LF (10 mg) was dissolved in 1 mL of the chosen solvent (Et_2_O, CH_3_CN or *n*-hexane) and maintained for about 20 h (overnight) at room temperature under magnetic stirring. After this time, the solvent was removed under a gentle flow of argon, and phosphate buffer at pH 7.5 (2.5 mL) was added to the residue. The suspension was maintained at 30 °C under vigorous magnetic stirring for 10 min, then compound **1** (21 mg, 0.17 mmol) was added, and TBHP (28 μL, 0.2 mmol) was dispensed within 10 min. The reaction course was monitored by HPLC as described above.

Series B: LF (10 mg) was dissolved in 2.5 mL of the solvent of choice (Et_2_O, CH_3_CN or *n*-hexane) and maintained for about 20 h (overnight) at room temperature under magnetic stirring. After this time, compound **1** (21 mg, 0.17 mmol) was added to the solution, and the reaction was started by dispensing TBHP (28 μL, 0.2 mmol) within 10 min. The reaction course was monitored by HPLC as described above.

### 3.8. Epoxidation of Fatty Matrix by LF-Associated Peroxygenase

LF (50 mg) was dissolved in 2.5 mL of the chosen solvent (phosphate buffer pH 7.5, Et_2_O, CH_3_CN or *n*-hexane) and maintained at 30 °C under magnetic stirring for 10 min, then TBHP (10 μL, 0.07 mmol) was added. After 4 h, the mixture was partitioned between water and Et_2_O, and the organic phase collected, dried on Na_2_SO_4_ and taken to dryness under a flow of argon. The residue was analyzed by ^1^H-NMR to detect the formation of epoxides of fatty matrix.

### 3.9. LF-Catalyzed Epoxidation of Limonene

A suspension of LF (50 mg) in 2.5 mL of the chosen solvent (buffer pH 7.5, Et_2_O, CH_3_CN or *n*-hexane) and *R*-limonene, **3** (9 μL, 0.055 mmol) was stirred at 30 °C for 10 min, then TBHP (15 μL, 0.11 mmol) was added in two aliquots over 30 min. The reaction mixture was left to react at 30 °C, and at appropriate times, aliquots (100 μL) were withdrawn for GC analysis. Samples in organic solvents were diluted with Et_2_O (200 μL), dried over Na_2_SO_4_ and injected. Samples in buffer were extracted with Et_2_O (200 μL), and the organic phase separated and dried over Na_2_SO_4_ before analysis. In spite of the larger mass of unreacted triglycerides (38 mg) and oxidized triglycerides (12 mg), limonene oxide **4** (7.8 mg, 93% isolated yield) was easily separated by chromatography (SiO_2_, *n*-hexane:Et_2_O 95:5).

### 3.10. Preparative Sulfoxidation of Thioanisole with LF

Procedure A: LF or MP (40 mg) was suspended in 2.5 mL of CH_3_CN and maintained for 10 min at 30 °C under magnetic stirring in a vial closed with the septum, then **1** (220 mg, 1.77 mmol) was added. The reaction was started by dispensing TBHP (300 μL, 2.2 mmol) with a syringe pump at a speed of 125 μL/h. The substrate conversion was monitored, withdrawing at suitable times aliquots (0.2 mL) of the reaction mixture, which was diluted with Et_2_O/MeOH 9:1 *v*/*v* (0.5 mL), dried on Na_2_SO_4_ and analyzed by chiral HPLC. The same conditions were also applied to reactions in *n*-hexane or CH_3_CN/*n*-hexane 20:80 *v*/*v* in the presence of LF.

In the solvent-free reaction, LF (40 mg) was dissolved in sulfide **1** (210 μL, 220 mg, 1.77 mmol) and the mixture stirred for 10 min at 30 °C in a vial closed with the septum. After this time, TBHP (300 μL, 2.2 mmol) was dispensed by syringe pump at of 80 μL/h rate. For monitoring substrate conversion, at appropriate times aliquots (20 μL) of the reaction mixture were withdrawn, diluted with Et_2_O/MeOH 9:1 *v*/*v* (0.7 mL), dried on Na_2_SO_4_ and analyzed by chiral HPLC.

Procedure B: LF (40 mg) was dissolved in 0.3 mL of CH_3_CN and maintained for 10 min at 30 °C under magnetic stirring in a vial closed with a septum. After this time, a 6.1 M solution of **1** in CH_3_CN and TBHP was simultaneously dispensed by two syringe pumps at a 70 μL/h rate.

For monitoring substrate conversion, at appropriate times, aliquots (20 μL) of the reaction mixture were withdrawn, diluted with Et_2_O/MeOH 9:1 *v*/*v* (0.7 mL), dried on Na_2_SO_4_ and analyzed by chiral HPLC. After 8 h reaction time, the final mixture was diluted with water (2 mL), extracted with Et_2_O (3 × 5 mL), and the organic layers collected and dried on anhydrous Na_2_SO_4_. The organic solvent was removed under reduced pressure, and the residue purified by chromatography on Silica gel column eluting with *n*-hexane/EtOAc 60:40 (*v*/*v*). Sulfoxide **2** (336 mg, 2.4 mmol, 70% yield, 84% *ee*) was obtained as a colorless oil with physical and spectroscopic properties in agreement with data in the literature [47].

## 4. Conclusions

In this work, the lipid fraction from oat flour was investigated for its peroxygenase activity as a more easily accessible alternative to the previously reported enzyme preparation from the same plant source. This new crude enzyme preparation showed good biocatalytic activity in the oxidation of thioanisole and the epoxidation of limonene. The peroxygenase embedded in the lipid fraction exhibited very high stability, even in pure organic solvents, probably associated with the presence of the fatty matrix. As evidence of this, a preparative-scale oxidation procedure for thioanisole was set up in CH_3_CN, and further studies are currently underway to better evaluate the potential of the developed enzymatic preparation in non-aqueous medium. In view of large-scale applications, the lower productivity metrics of a crude enzyme preparation compared to a purified enzyme might be balanced by its cost-effectiveness, and a focused study is obviously necessary. The obtained results could also pave the way for the investigation of enzymatic activities associated with the lipid fraction of other plant seeds.

## Data Availability

The original contributions presented in this study are included in the article/Supplementary Materials. Further inquiries can be directed to the corresponding author.

## Supplementary Materials

The supporting information can be downloaded at: https://www.mdpi.com/article/10.3390/ijms26199431/s1.

## Abbreviations

The following abbreviations are used in this manuscript:

LF	Peroxygenase-containing Lipid fraction from oat flour
MP	Peroxygenase-containing Microsomal Preparation from oat flour
TBHP	*Tert*-butyl hydroperoxide
t-BME	*Tert*-butyl methyl ether
