# Supplementary material for: Use of Lipid Extract of Oat Flour as a Peroxygenase-Containing Biocatalyst Active in Organic Solvents
Source: Int J Mol Sci. 2025 Sep 26;26(19):9431. doi: 10.3390/ijms26199431 (PMC12524458; doi:10.3390/ijms26199431)
Supplement: Supplementary file 1 [file ijms-26-09431-s001.zip › ijms-3833035-supplementary.pdf]

Use of lipid extract of oat flour as a peroxygenase-containing biocatalyst active in organic solvents

Claudia Sanfilippo and Angela Patti*

Institute of Biomolecular Chemistry – National Research Council of Italy, Via Paolo Gaifami 18, I-95126 Catania, Italy

Correspondence: angela.patti@cnr.it

Supporting information

	Page
**Figure S1.** Appearance of peroxgenase-containing lipid fraction from oat flour.	S1
**Figure S2.** Representative ^{1}H-NMR spectrum of lipid fraction from oat flour	S1
**Figure S3.** ABTS assay on lipid fraction obtained by extraction of oat flour with different solvents.	S2
**Figure S4.** Light microscopy images of LF suspended in buffer	S2
**Figure S5.** ABTS assay monitoring of LF stability.	S3
**Figure S6.** Oxidation of thioanisole different reaction media by using LF-enzymatic preparation preincubated for 20 h in different organic solvents.	S3
**Figure S7.** TLC analysis of (left) LF and (right) LF after epoxidation by LF-associated peroxygenase in phosphate buffer (4h).	S4
**Figure S8.** Ratios between methyl and ethylenic protons in (A) crude lipid fraction from oat flour and (B) after epoxidation (4 h) by LF-associated peroxygenase in phosphate buffer.	S4

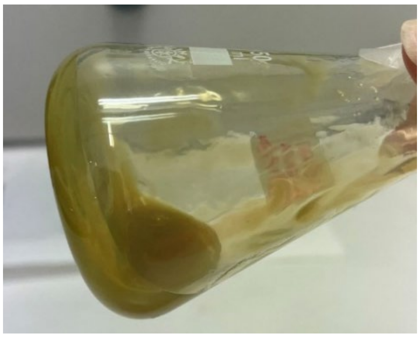

Figure S1. Appearance of peroxgenase-containing lipid fraction from oat flour

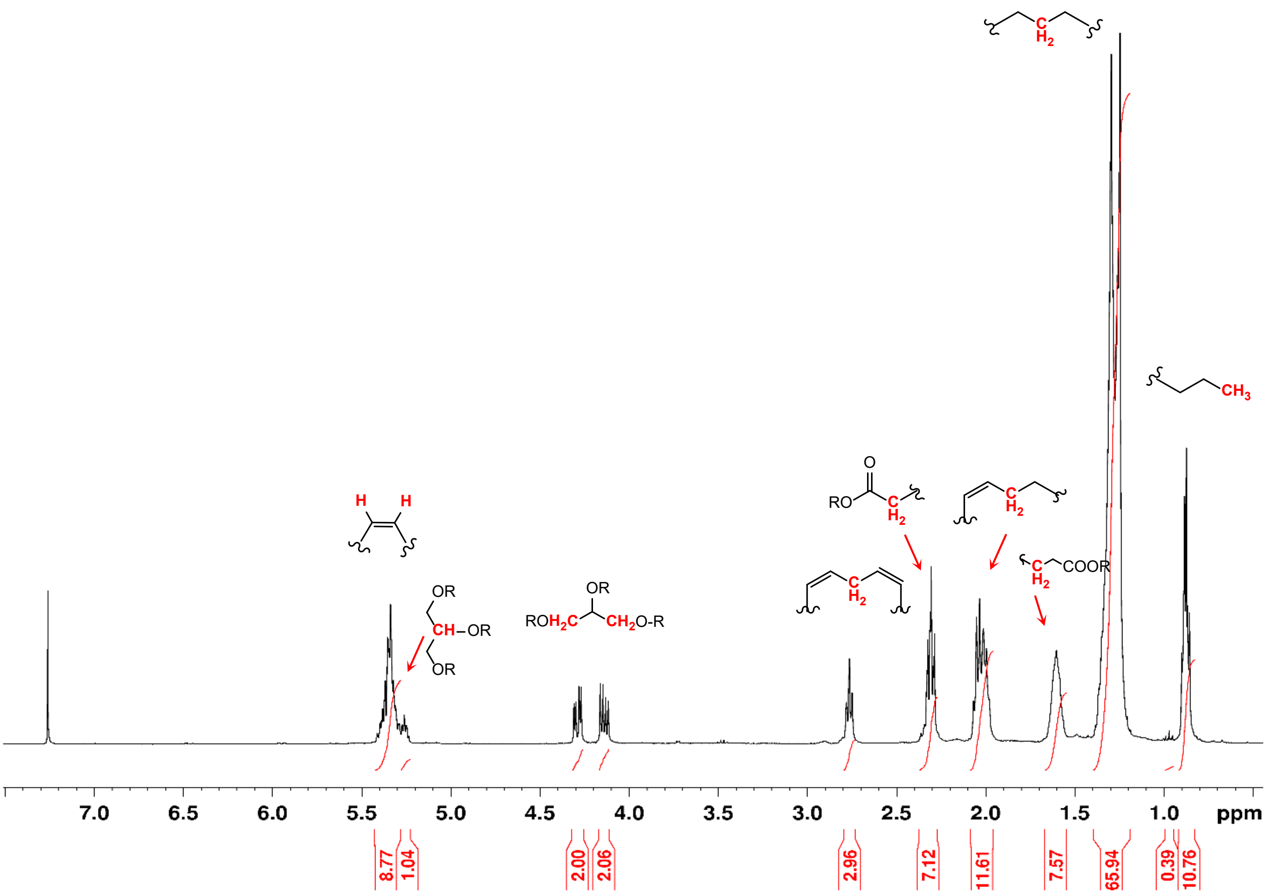

Figure S2. Representative ^{1}H-NMR spectrum (in $CDCl_3$) of lipid fraction from oat flour

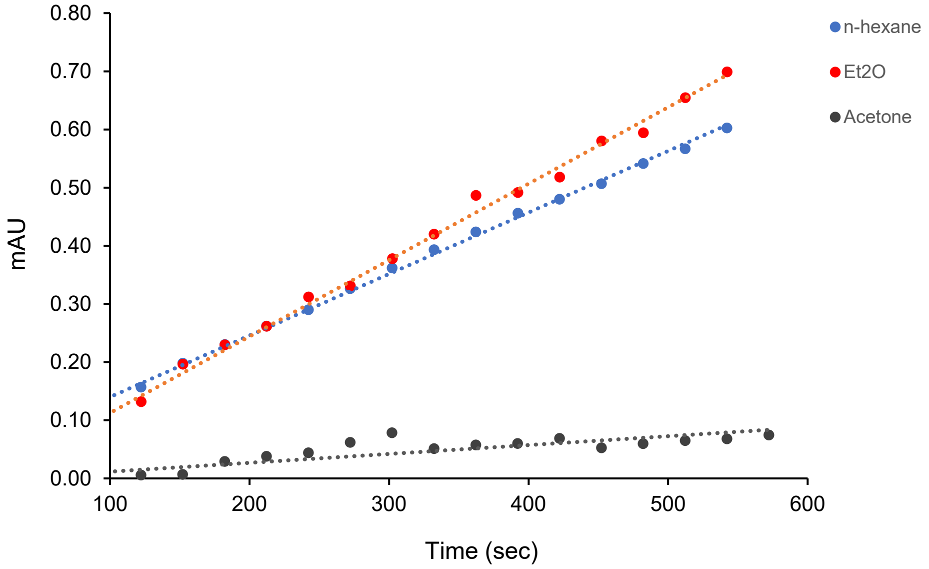

Figure S3. ABTS assay on lipid fraction obtained by extraction of oat flour with different solvents.

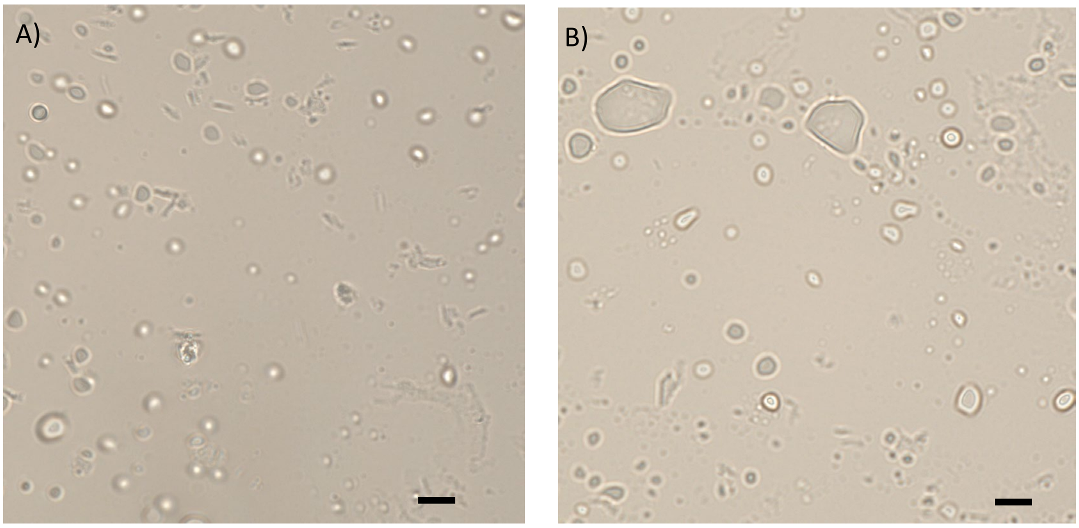

Figure S4. LF suspended in (A) pH 7.5 buffer and (B) pH 6.5 buffer. Bar represents 10 µm.

.

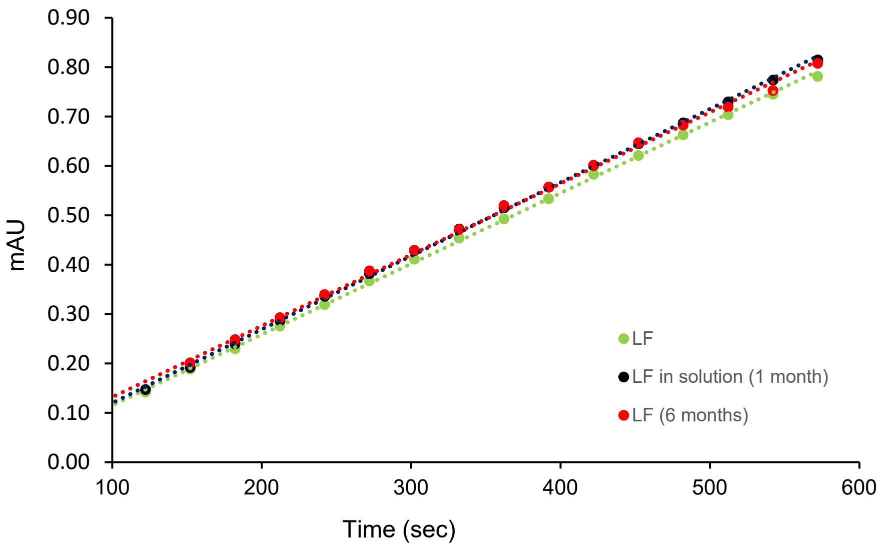

Figure S5. ABTS assay monitoring of LF stability.

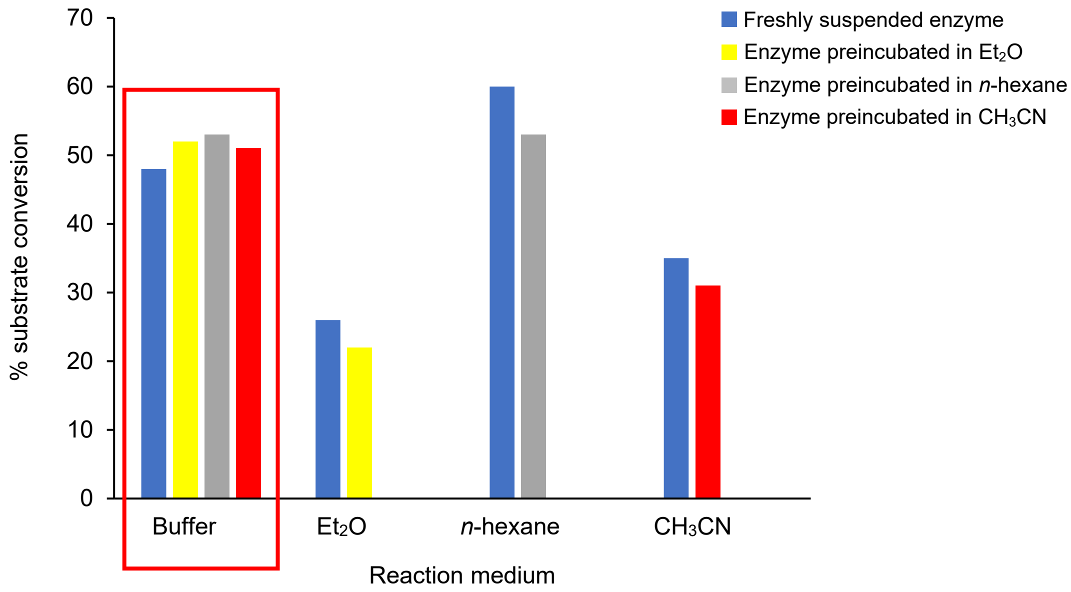

Figure S6. Oxidation of thioanisole different reaction media by using LF-enzymatic preparation preincubated for 20 h in different organic solvents (after 10 min reaction time).

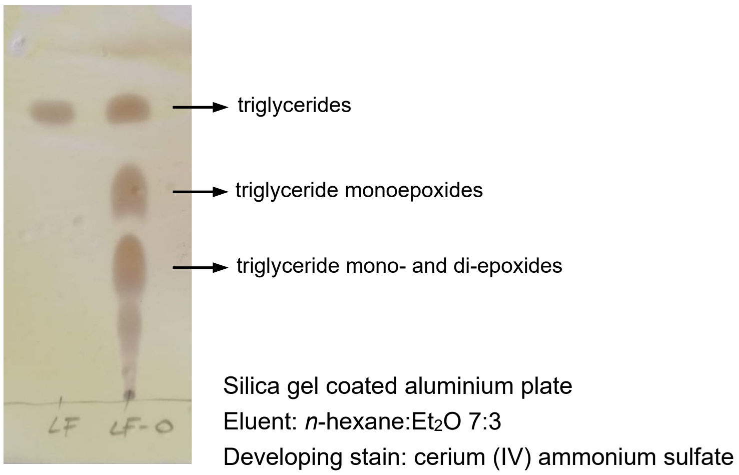

Figure S7. TLC analysis of (left) LF and (right) LF after epoxidation by LF-associated peroxygenase in phosphate buffer (4 h).

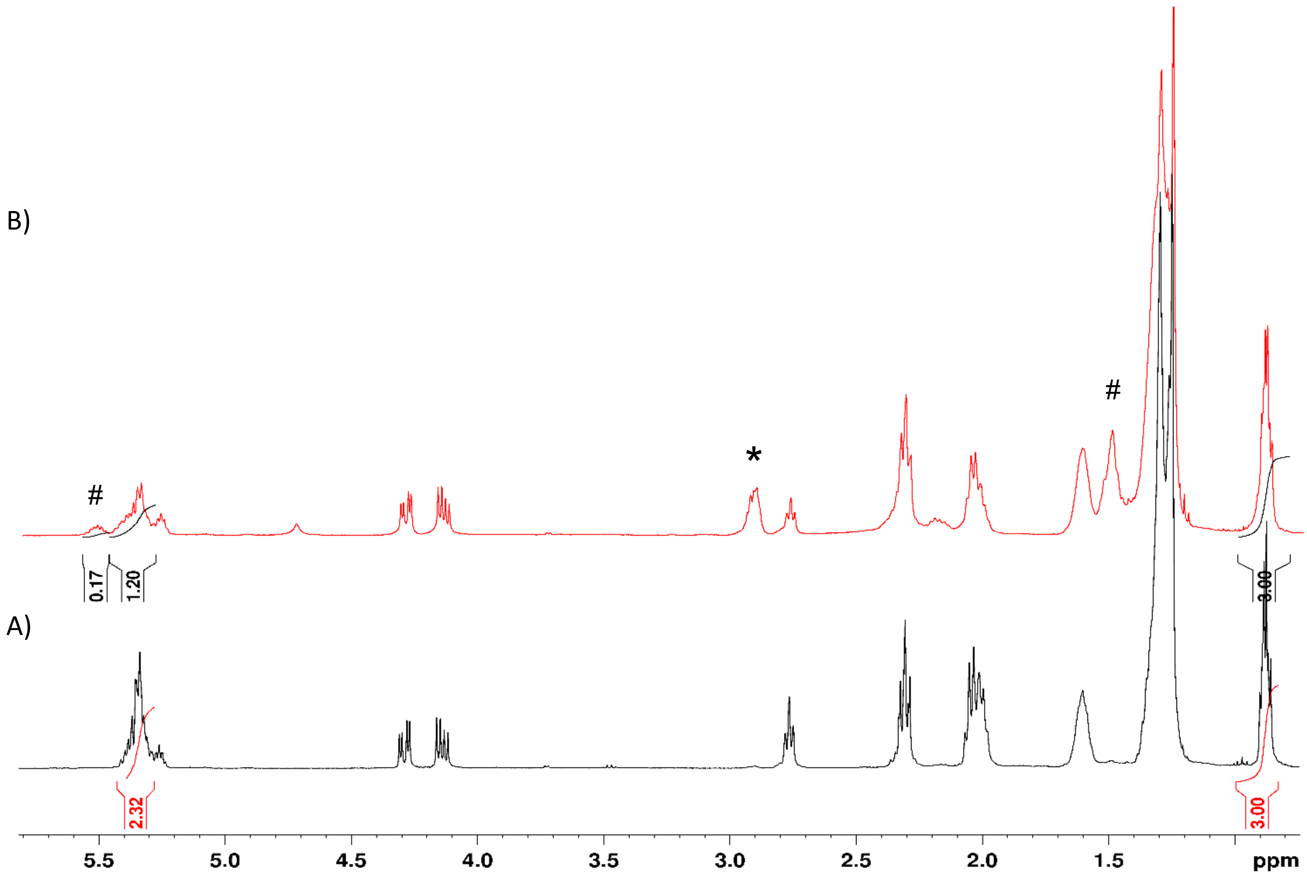

Figure S8. Ratios between methyl and ethylenic protons in (A) crude lipid fraction from oat flour and (B) after epoxidation (4 h) by LF-associated peroxygenase in phosphate buffer. * = resonance associate to epoxide moiety; # = shifted resonances of protons in acyl chains following epoxidation.
